# Inferring plant-bee-microbe associations: Foragers, hive workers, and honey tell complementary stories

**DOI:** 10.1371/journal.pone.0351230

**Published:** 2026-07-08

**Authors:** Jordan Twombly Ellis, Alyssa R. Cirtwill, Emilie E. Ellis, Tomas Roslin, Helena Wirta

**Affiliations:** 1 Department of Ecology, Environment and Geoscience, Umeå University, Umeå, Sweden; 2 Carex EcoLogics, Bracebridge, Ontario, Canada; 3 Research Centre for Ecological Change, Organismal and Evolutionary Biology Research, University of Helsinki, Helsinki, Finland; 4 Ecosystems and Environment Research Programme, Faculty of Biological and Environmental Sciences, University of Helsinki, Helsinki, Finland; 5 Department of Ecology, Swedish University of Agricultural Sciences, Uppsala, Sweden; 6 Department of Agricultural Sciences, Faculty of Agriculture and Forestry, University of Helsinki, Helsinki, Finland; Tanta University Faculty of Science, EGYPT

## Abstract

Pollinators, both wild and managed, form diverse associations with plants and microbes which affect the wellbeing of the plants and the pollinators. The method by which these associations are sampled impacts our understanding of the system. The common ways to understand pollinator-plant or pollinator-plant-microbe associations are to observe flower visits of insects, or to collect foraging individuals and identify the pollen and microbes they carry. Honey bees offer a test case for methods of sampling these associations. Hives of managed honey bees host thousands of pollinator individuals together with jointly-collected nectar which is turned into honey. Previous studies have used DNA preserved in honey to infer honey bee associations with plants and microbes. Here, we sampled honey, individual bees while they were foraging, and groups of bees from inside the hive. We identified plants and microbes on the surface of the bees or in the honey using DNA metabarcoding – expecting that bees sampled singly or in groups would reveal a subset of the associations recorded in the communal honey deposits. However, we found that each sample type revealed different aspects of the richness and community composition of plants and microbes encountered by bees. Both honey samples and hive bees had more plant and microbial taxa per sample than samples of individual bees. Though individual bees are subsets of the larger colony, pollen and microbe associations recovered from individual bees did not represent a subsample of associations recovered from groups of hive bees or from honey. Thus, while each sampling technique provides information about honey bee ecology, they are not equivalent. DNA in honey represents time-integrated associations between bees and the surrounding ecosystem; the hive bees provide a snapshot of current colony-level associations, and individual foraging bees capture intraspecific variation in foraging preferences and microbe exposure.

## Introduction

Pollination is an important ecosystem service, with an estimated 75% of the reproductive success of flowering plants worldwide being enhanced by, or dependent upon, pollinators [1,2]. Understanding the associations of pollinators and the flowering plants they choose to visit, and may thereby pollinate, is crucial to safeguard the pollination of crops and wild plants [3] but is challenging due to the difficulty of observing these interactions directly. Historically, reconstructions of plant-pollinator communities most commonly comprised records of insects visiting particular flowers [4]. More recently, plant-pollinator associations have also been inferred by DNA metabarcoding of pollen recovered from the surface of collected insects [5–7]. The latter approach will generally reveal many associations per individual insect, providing insight into variability in associations within insect species [8]. Moreover, DNA metabarcoding also reveals insects’ associations with taxa other than plants.

In particular, microbes are an unseen but important third partner in plant-pollinator interactions [9]. Bacteria and fungi are found throughout the ecosystem, including on the surface of and inside pollinators and flowers [10]. These microbial communities are shaped by insect visits to flowers as pollinators can deposit and acquire microbes during each flower visit. Microbes transferred by pollinators can be beneficial, pathogenic, or commensal to pollinators and plants [11,12]. Certain microbes can alter the attractiveness of nectar to pollinators, thus modifying the associations between plants and pollinators [13]. Therefore, the transmission of microbes via flower visits is important for all parties: plants, pollinators, and the microbes themselves [14].

Among managed pollinators, honey bees (*Apis mellifera*) are the most economically important, providing hundreds of billions of dollars’ worth of pollination services per year worldwide [15,16]. Being widespread generalist foragers with colonies of tens of thousands of individuals [17], honey bees can be efficient pollinators throughout the ecosystem [18]. At the same time, they act as important microbe transporters. Honey bees harbor diverse individual and colony microbiomes [19,20]. Among these, many honey bee-associated pathogens can be transmitted to flowers and then to other pollinator hosts, and vice versa [21,22]. Honey bees acquire their core gut microbes shortly after emergence from pupation via interacting with other colony members [23] but also acquire microbes from the flowers they visit and from their environment more generally [24]. Microbes (and pollen) carried on honey bee surfaces or in the gut can be trapped in honey during its production, hence honey stored in the hive provides a record of the plants and microbes honey bees have encountered while foraging, without needing to capture individual bees in the field [25–27].

Despite the promise of honey as an efficient sampling method to detect associations between honey bees and plants or microbes, it is not yet clear whether this approach is comparable to more traditional methods such as direct observations of flower visits by individual insects [4] or identifying the pollen carried on the surface of singly-collected pollinator individuals [28,29]. Comparisons of visitation observations and body-surface pollen identification show that the sampling approach can strongly affect our perception of what associations occur [8,30,31]. For wild pollinators sampled in the field, individual-based sampling is typically the only option, making it difficult to assess how well the ecological associations reconstructed from individuals represent the entire community. Yet, for managed honey bees kept in hives, other sampling methods are available as points of comparison. Some studies collect bees directly from the inside of hives [20,32]. Others use honey samples as a colony-level proxy for what plants and microbes honey bees are collecting from the ecosystem [25,27,33,34]. In order to compare studies based on these different approaches, we need to know whether and how the sampling approach taken affects the associations detected.

In this study, we compare the plant, bacteria, and fungi associations detected in each of the above sample types (individual bees collected while foraging, bees collected in groups from inside the hive, and honey sampled from individual hives). All associations were identified by DNA metabarcoding the pollen and microbes carried on honey bee surfaces or in honey. We hypothesize that individual bees, each making a small contribution to the hive as a whole, contact only a subset of the pollen and microbes contacted by the hive and stored in honey. Further, we expect that the associations with pollen and microbes found on a group of hive bees represent a random subsample of the associations archived in honey. We therefore expect honey to provide the most thorough record of plants and microbes encountered by bees, followed by groups of bees sampled from the hive and then individual bees. We expect these trends to be consistent across plants, bacteria, and fungi.

## Materials and methods

### Sample collection

To examine the impact of sampling type on the associations found, we collected three types of samples: individual foraging bees, pooled samples of bees from the hive, and honey, in Southwest Finland (62° 27’ N, 22° 58’ E) from 15^th^ to 17^th^ of June 2023. This agricultural region is a mosaic of fields and different sized patches of boreal coniferous forest. To sample foraging bees, hive bees and honey, we used five apiaries, three by crop fields and two in forests. We established study sites 100m and 800m from the selected apiaries and marked two transects of 2 * 25 m, equaling 100 m^2^ in each site. To describe the flowering plant community available for the bees in the area, we identified all the flowering plants within the transects.

Foraging bees were collected on these transects by netting them individually in clean plastic bags to avoid cross-contamination between individuals. These bees were collected when they were visiting flowers and were stored immediately on dry ice. To allow comparison to direct observations of flower visits, we also recorded the species of flower on which foraging bees were caught. Hive bee and honey samples were collected from hives maintained by the only beekeeper in the area. Thus, the foraging bees collected originated from the same apiaries that we collected hive bees and honey from.

Honey and hive bees were sampled from 19 colonies across the five apiaries. Pooled samples of hive bees were sampled from colonies by opening the hive and quickly scooping 6–25 bees into a DNA-free 50 ml tube (Eppendorf, Germany) before the bees could fly away and were stored immediately on dry ice. This provided a representation of adult bees of various ages. Honey samples totaling approximately 50 ml were collected from these same hives with DNA-free spoons (sterilized by heating for 5 hours in 200°C) from three different frames into DNA free 2 ml tubes (Eppendorf, Germany). We collected only new, recently-covered honey representing nectar collected during approximately the last 7–10 days [35,36].

Foraging bees were transferred into DNA free 2 ml tubes with DNA-free forceps, and both the foraging bee and hive bee sample tubes were filled with 99.5% ethanol the same day they were collected. All samples were stored in a −20ºC freezer until further processing. The beekeeper whose colonies we sampled had agreed to us collecting both honey and bees from her hives. Further, in Finland no research permits are required for collecting insects.

### Sample preprocessing

From the surface of singly collected foraging bees, we detached microbes and pollen by sonicating the closed tubes in a water bath for 5 minutes. The samples were then vortexed at full speed for 2 × 10 seconds. As a result, the microbes and the pollen were suspended in the ethanol within the tube. Afterward, each bee was carefully removed from the tube using DNA-free forceps, leaving the detached pollen and microbes in the solution. The samples were centrifuged at 11 000 xg for 10 minutes and the remaining supernatant was removed. To let all the excess ethanol evaporate, the tubes were left open and covered with clean tissues and kept at 60°C for 60 minutes. The bee samples collected from the hives were treated the same as the samples of forager bees, until the centrifugation step when sample tubes were centrifuged at 8000 xg for 60 minutes, after removing the bees (Centrifuge 5810 R, Eppendorf, Germany). In the next step, most of the supernatant was discarded and the pellet was transferred to a 2 mL tube. Finally, the pollen-microbe sample in the 2 mL tube was further processed as for foraging bees.

For the honey samples, 10 g of honey was diluted to 30 mL with DNA clean water (MilliQ, Merck KGaA, Germany) in a 50 mL tube. The honey was dissolved into the water for 30 minutes at 60°C. The samples were then centrifuged at 8000 xg for 60 minutes (Centrifuge 5810 R, Eppendorf, Germany), after which most of the supernatant was discarded and the pellet was transferred to a 2 mL tube. The 2 mL tube was further centrifuged at 11 000 xg for 5 minutes and the remaining supernatant was removed. All the preprocessed samples were stored in a −20ºC freezer until DNA extraction.

### DNA metabarcoding

We identified the taxonomic origins of the plant, bacterial, and fungal DNA in all the samples using DNA metabarcoding. To this aim, we amplified parts of ITS2 regions for plants and fungi, and parts of 16S for bacteria, adopting the same procedures for DNA laboratory analyses, sequencing, and bioinformatic processing of reads as done by Tiusanen et al. [20]. After preprocessing the samples, DNA was extracted with the DNeasy Plant Mini Kit (Qiagen, Germany). The targeted fragments of the specific gene regions were first amplified with tagged primers ITS2-F and ITS2-R [37,38] for plants; 16S_515FB and 16S_806RB [39,40] for bacteria; and ITS3-KYO2 and ITS4-KYO3 [41] for fungi. The original sample was split into two replicates, with identical PCR reactions performed for both. The replicates were combined for the 2nd PCR to attach unique combinatorial indices to each sample. For each batch of samples, negative control samples were included at the DNA extraction step. In the taxon and gene region specific PCRs, we added a second set of negative controls using DNA clean water (MilliQ, Merck KGaA, Germany). All negative controls were indexed the same way as the other samples.

All the samples were combined for sequencing and sequenced with Illumina MiSeq using v3 chemistry with 600 cycles and 2 x 300 bp paired-end read length. For the bioinformatics processing, the reads of all samples were combined per gene region. The reads were truncated, merged and quality controlled. Primers were removed, the reads were dereplicated, and singletons were removed. The reads were denoised to zero-radius operational taxonomic units (ZOTUs) [42]. To remove possible misassigned reads and false positives, due to contamination, the reads in ZOTUs were filtered as follows: The maximum number of ZOTU-specific reads found in the DNA extraction or the PCR negative controls was subtracted from the sample. If that resulted in zero reads, then the ZOTU was deleted from the sample.

For plants, the taxonomic assignment of ZOTUs was done by comparison against an ITS2 reference database from PLANTiTS [43], accessed 21.3.2022. For bacteria, sequences were compared against the 16S RDP reference database [44], version 18, and for fungi against the UNITE fungal ITS reference database [45], version 10.05.2021. Taxonomic assignments were accepted with the threshold 0.9 of SINTAX probability [46]. Families and genera for which the average relative read abundance across the samples was less than 0.1% were omitted. Bees were considered to have had an association with microbes that were either found on their surface or in a honey sample. Similarly, bees were considered to have had an association with plants if their pollen was found on the pollinator’s surface or in honey samples.

### Statistical analysis

To establish whether the sample type impacts which associations are detected, we compared the three sample types in regard to the richness, community composition and identity of the plants, bacteria and fungi detected from each sample type. In all analyses, we used R version 4.5.0 [47]. We present results from presence-absence data unless analyses are otherwise clarified to have been done using relative read abundance data. We first tested whether there were relationships between log read depth and the number of associated taxa using negative binomial generalized mixed models (fit using the function ‘*glm.nb*’ from the package *stats* 3.6.2 [48]. We fit separate models to each of the following five response variables: the count of plant ZOTUs; the count of plant genera; the count of bacterial ZOTUs, the count of bacterial genera; and the count of fungal ZOTUs. To determine whether the sample types differ in the number of taxa detected per sample for each sample type, we used ANOVA and Tukey’s HSD post-hoc tests in package *stats* 3.6.2. To visualize the community composition of plants, bacteria, and fungi detected within different sample types, we performed principal component analyses (PCA) using the function *‘prcomp’* in package *stats* 3.6.2. To compare beta dispersion of the communities in each sample type, we used the function “*betadisper*” and compared community centroids (analogous to a multivariate average) using a PERMANOVA test fit with the function “*adonis2*” (999 permutations). Both tests were performed in package *vegan* 2.7−1 [49]. These analyses were done using both presence-absence matrices as well as relative read abundance matrices. To compare the proportions of plant, bacterial and fungal taxa unique to vs. shared between sample types, we constructed Euler diagrams with package *eulerr* 7.0.2 [50]. We then investigated the percentage of relative reads that these unique ZOTUs comprised to contextualize how relatively common they were.

### Subsampling analysis

To determine whether the plants and microbes found on individual foraging bees represent a random subsample of those found in the pooled samples (hive bees or honey), we resampled the latter two datasets. To this aim, we first assigned a number of ZOTUs, *z,* to each virtual forager bee. The value of *z* was drawn from the set of ZOTU counts observed among real individual foraging bees. We then randomly sampled *z* ZOTUs without replacement, with the probability of selecting any given ZOTU set by the ZOTU counts in the *group* being sampled from. This exercise was repeated separately for plants, bacteria, and fungi, with the subsampled *group* defined as hive bees and honey, respectively. For each iteration, we created as many virtual bees as real bees observed (with 31 bees scored for plants, 26 for bacteria, and 21 for fungi). We then tested whether the microbial community of the virtual foragers differed from that of the real foragers. For this, we used a PERMANOVA with 1000 permutations, fit using the function *‘adonis2’* [49]*.* We repeated this process 1000 times, then plotted the R^2^ values from each PERMANOVA test of each simulation. To determine whether the distributions significantly differed from each other, we used a two-sample t-test.

To determine whether the plants and microbes found on hive bees represented a random subsample of those found in honey, we repeated the process above. In this case, the target number of ZOTUs, *z*, was set by the distribution of ZOTU richness in samples of hive bees, and the group drawn from was defined by the ZOTU pool observed in honey. Statistical significance was determined using the same approach as above.

## Results

We collected 31 individual foraging bees, 19 pooled hive bee samples, and 18 honey samples (one of the sampled colonies had no recently-covered honey at the time of sampling) and examined the plant, bacterial, and fungal DNA found in honey and on the surfaces of foragers and hive bees. We obtained plant reads for all samples and fungal and bacterial reads for all hive bee and honey samples, but for only 21 and 26 of the foraging bee samples respectively. For plants and bacteria, the majority of reads could be assigned to the taxonomic level of genera (Table 1). All samples yielded plant reads assigned to genus, while 26 out of 31 foraging bee samples yielded bacterial reads that could be assigned to genus. A far smaller proportion of fungal reads could be assigned to family or genus across all the samples. Therefore, for all three taxonomic groups, we report results based on ZOTUs in all analyses. When explicitly comparing genera detected in different sample types, we exclude fungi to avoid biased interpretation due to poor identification to genera of most fungal DNA. As read counts were relatively low for our samples (Table 1), samples with greater numbers of reads tended to have more bacterial and fungal ZOTUs and bacterial genera. However, there was no relationship between read counts and number of plant ZOTUs or genera (S1 Fig and S1 Table).

**Table 1:** Average number of reads per sample, with standard deviation, and the resulting percentage of reads assigned to family and genus for plants, bacteria, and fungi.

	Avg. reads per sample (±SD)	Proportion of reads assigned to family	Proportion of reads assigned to genus
Plants	5356 (±1963)	99.6%	90.0%
Bacteria	3341(±3068)	93.8%	90.0%
Fungi	3631 (±4463)	42.5%	38.4%

**Fig 1:**
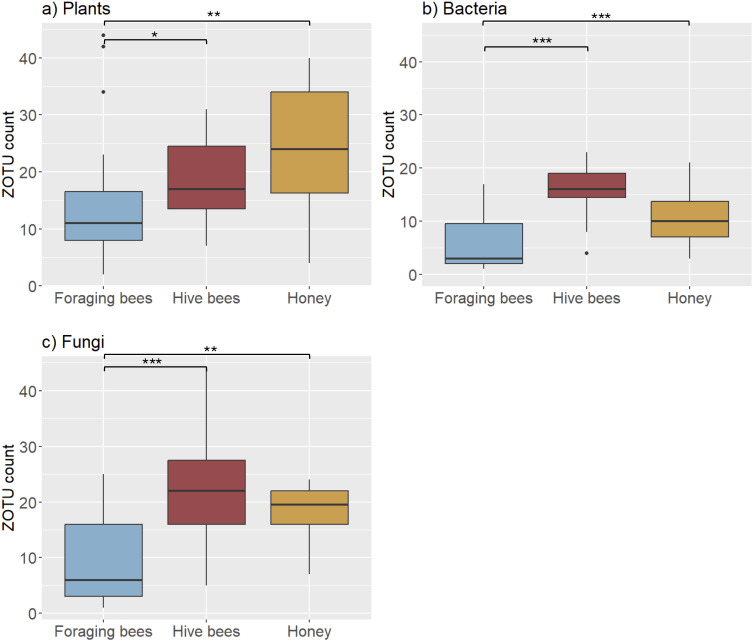
Average number of ZOTUs per sample found in different sample types across plants (a), bacteria (b), and fungi (c). Foraging bee samples consistently yielded the fewest ZOTUs per sample. Foraging bees are represented in blue, hive bees in red, and honey in yellow. Significance is shown with asterisks, *p*-value < 0.05 = *, < 0.01 = **, < 0.001 = ***.

The mean number of plant ZOTUs per sample significantly differed between sample types (Fig 1 and Table 2). Specifically, honey samples and hive bees tended to yield more plant ZOTUs (21.4 ± 10.1 and 18.6 ± 6.72, respectively) than individual foraging bees (14.3 ± 10.3). For bacteria, all three sample types had significantly different mean numbers of ZOTUs per sample, with the foraging bees yielding the lowest bacterial richness (5.67 ± 5.20) and the hive bees the highest (15.7 ± 4.74), with the richness recovered from honey falling in between (10.3 ± 5.40). For fungi, both honey and hive bees had higher mean numbers of ZOTUs per sample (18.4 ± 4.96 and 22.4 ± 9.70, respectively) than individual foraging bees (8.67 ± 7.65). These trends remained similar when we examined the richness of genera found with each sample type. Samples of individual foraging bees yielded significantly fewer genera of plants and bacteria than samples of hive bees or honey (S2 Table). Hive bees were pooled samples that contained between 6 and 25 bees per sample. However, there was no significant relationship between number of hive bees per sample and number of plant, bacterial, or fungal ZOTUs per sample (S2 Fig).

**Table 2:** Results of ANOVA and Tukey Post hoc test on comparisons of log-transformed average number of ZOTUs found from the different sample types of individual foraging bees, hive bees, and honey across plants, bacteria, and fungi. Significant *p*-values are shown in bold.

	ANOVA	Comparison	Tukey HSD *p*-value
Plants	F_2_ = 7.181 *p =* **0.002**	Foraging bees vs. hive bees	**0.044**
		Foraging bees vs. honey	**0.002**
		Hive bees vs. honey	0.537
Bacteria	*F*_*2*_ = 19.83 *p* **< 0.001**	Foraging vs. hive bees	**<0.001**
		Foraging bees vs. honey	**<0.001**
		Hive bees vs. honey	0.109
Fungi	*F*_*2*_ *=* 19.95 *p* **< 0.001**	Foraging vs. hive bees	**<0.001**
		Foraging bees vs. honey	**<0.001**
		Hive bees vs. honey	0.839

**Fig 2:**
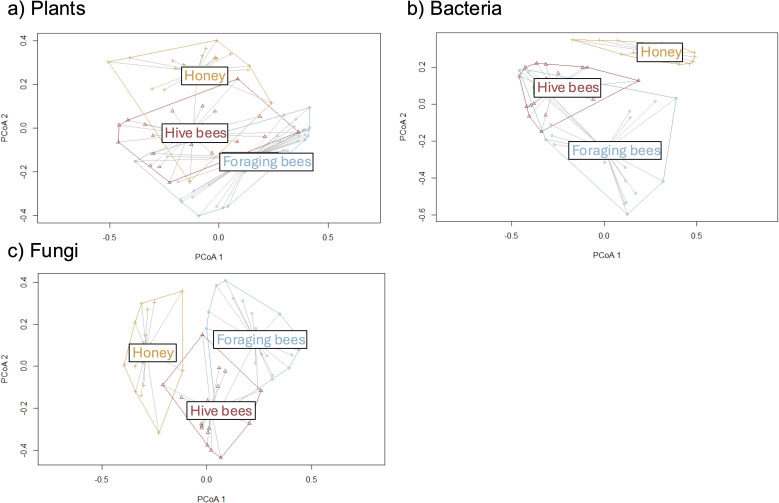
Variation in the community composition of plant (a), bacterial (b), and fungal (c) ZOTUs based on principal component analysis (PCA) of the distance of the dispersion of communities from the centroid. Community composition of plants and microbes found on foraging bees varies more than those found on hive bees and in honey, with the exception of the community composition of plants detected on hive bees (statistics shown in Table 3). Foraging bees are represented in blue, hive bees in red, and honey in yellow.

Variation in community compositions of plant, bacterial, and fungal ZOTUs differed among all sample types (Fig 2). For plants, the average distance to the centroid of different sample types showed greater dispersion in samples of foraging bees (0.500) than honey (0.374), whereas the dispersion of the individual foraging bee samples did not differ from that among samples of hive bees (0.465) (Table 3). For bacteria, individual foraging bees (0.559) showed the largest dispersion compared to hive bees (0.273) and honey (0.457) (Table 3). Similarly, fungal communities found on individual foraging bees (0.644) had a larger dispersion than hive bees (0.488) or honey (0.355). In other words, individual foraging bees showed the most variable composition of plant, bacterial, and fungal ZOTUs among samples as compared to the other two sample types. This same analysis done using relative read abundance found similar results. Plant communities found on foraging bees had significantly different dispersions to hive bees, bacterial communities had significantly different dispersions in foraging bees compared to honey, as well as in hive bees compared to honey. All sample types were found to have significantly different dispersions of their fungal communities (S3 Fig and S4 Table).

**Table 3:** Average distance to centroid of plant, bacterial, and fungal ZOTUs from each sample type. Dispersion p-values refer to pairwise tests for differences in dispersion between each pair of sample types, whereas significance values for PERMANOVA tests refer to differences between centroids.

	Comparison	Dispersion *p*-value	Dispersion F-stat	PERMANOVA *p*-value
Plants	Foraging vs. hive bees	0.375	F-stat 4.992	*p*-value **0.001** F-stat 9.029
	Foraging bees vs. honey	**0.012**		
	Hive bees vs. honey	**0.012**		
Bacteria	Foraging vs. hive bees	**0.002**	F-stat 38.388	*p*-value **0.001** F-stat 14.773
	Foraging bees vs. honey	**0.003**		
	Hive bees vs. honey	**0.002**		
Fungi	Foraging vs. hive bees	**0.002**	F-stat 50.31	*p*-value **0.001** F-stat 7.635
	Foraging bees vs. honey	**0.002**		
	Hive bees vs. honey	**0.002**		

At the level of bacterial genera, individual foraging bees again showed the widest dispersion of bacterial genera. For plant genera, we detected no significant differences between sample types (S4 Fig and S3 Table). Different sample types also exhibited significantly different community centroids (Table 3). Although significantly different dispersions can affect the reliability of tests for different centroids, in our case the greater dispersion of the foraging bees (the largest group of samples) would lead the PERMANOVA test to be overly conservative [51]. Therefore, we are confident that the community centroids are truly different.

**Fig 3:**
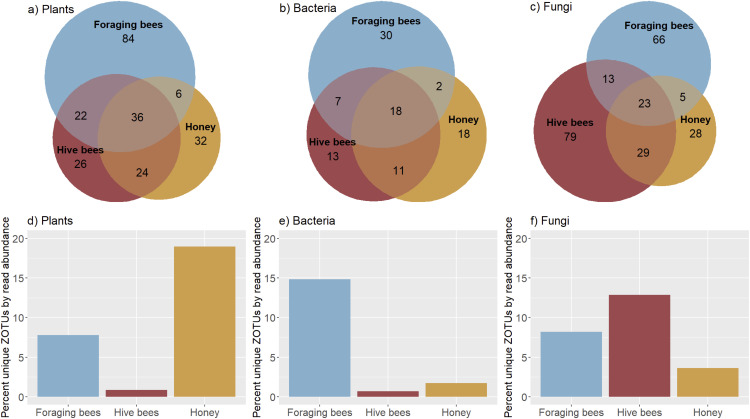
Number of unique and shared plant (a), bacterial (b), and fungal (c) ZOTUs across the different sample types. All sample types showed unique ZOTUs, with similar amounts of ZOTUs shared between types. Foraging bees are represented in blue, hive bees in red, and honey in yellow. The percentage of total reads made up of unique plant **(d)**, bacterial **(e)**, and fungal **(f)** ZOTUs for each sample type. Illustrating if unique ZOTUs are relatively rare or common based on read abundance data.

Despite the partial overlaps in community composition, each sampling method detected many ZOTUs unique to each taxonomic group (Fig 3). For plants, individual foraging bees carried the largest percentage (36.5%) of unique ZOTUs, while 11.3% of plant ZOTUs were unique to hive bees and 13.9% unique to honey (Fig 3a). These unique ZOTUs made up 7.8% of total plant reads found on foraging bees, 0.88% of reads found on hive bees and 19% found in honey (Fig 3d). For bacteria, all three sample types yielded similar numbers of ZOTUs, of which 30.3% were unique to individual forager bees, 13.1% to hive bees, and 18.1% to honey (Fig 3b). Unique ZOTUs comprised 15%, 0.67%, and 1.7% of total bacterial reads for each sample type, foraging bees, hive bees, and honey respectively (Fig 3e). For fungi, hive bees showed the largest percentage of unique ZOTUs (32.5%), while 27.2% were unique to foraging bees, and 11.5% to honey (Fig 3c). The unique fungal ZOTUs found from each sample type made up 12.9%, 8.2%, and 3.6% of total reads for hive bees, foraging bees, and honey respectively (Fig 3f).

For DNA reads identified to genera, all three sample types revealed genera unique to individual sample types. Each sampling method yielded a similar number of unique plant genera (21.4% unique to foraging bees, 14.3% unique to hive bees, and 7.14% unique to honey). Likewise, for bacterial genera, 32.5% were unique to foraging bees, 12.5% to hive bees, and 22.5% to honey; S5 Fig).

**Fig 4:**
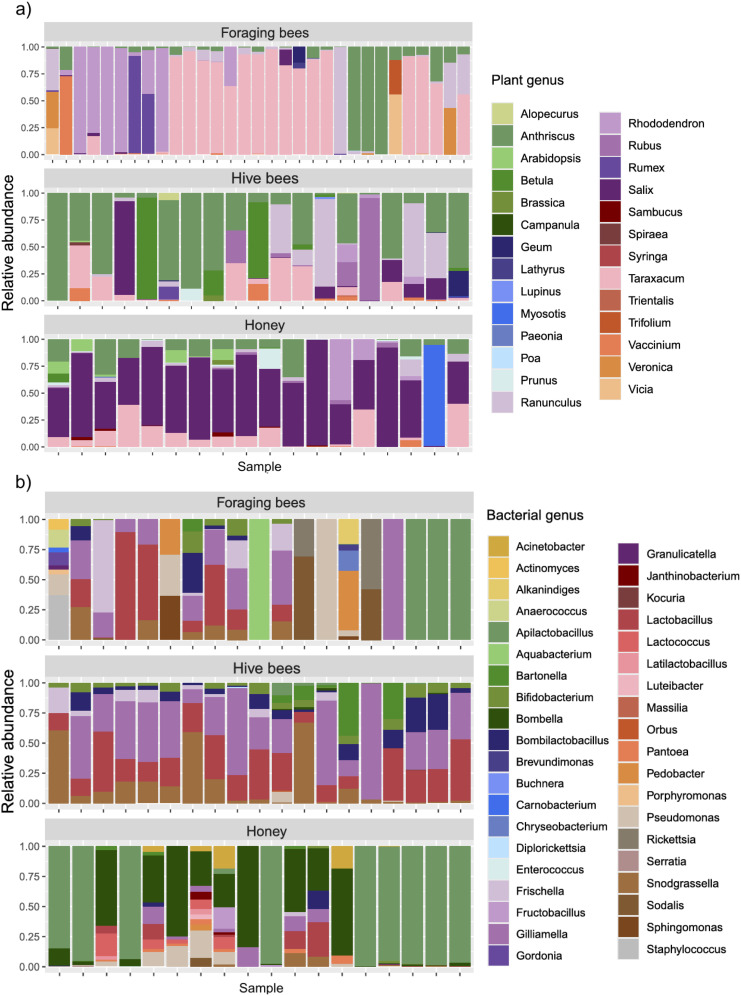
Stacked bar plots of a) plant and b) bacterial genera across sample types displayed by the relative read abundance of each genus. Plants such as *Arthriscus* (muted green) and *Taraxacum* (pink) were common across all three sample types but predominant in hive bees and foraging bees relatively, while *Salix* (dark purple) was found mainly in honey samples. Among bacteria, hive bees had a lot of gut bacteria such as *Lactobacillus* (red), *Gillamella* (purple), and *Snodgrassella* (light brown) while foraging bee and honey samples included a wider variety of bacteria with a notable high relative abundance of *Apilactobacillus* (light green).

Comparing the relative read abundance of genera found in each individual sample revealed considerable variation within and between sample types (Fig 4). Two locally common plant genera that were observed in our flowering vegetation counts, *Anthriscus* and *Taraxacum*, (0.18 and 0.31 relative read abundances respectively) were common across sample types, and the individual foraging bees were also observed visiting both genera during sampling (Fig 4, S5 and S6 Tables). The genus *Salix* was found in most honey samples but only rarely in the two types of bee samples and was observed at low abundances in flowering vegetation counts (S6 Table). Differences in bacteria between sample types were much starker. Despite only sampling bee surfaces, the hive bee samples showed quite a few bacterial genera characteristic of the honey bee gut microbiome, such as *Lactobacillus* and *Gilliamella*, that were not as frequent nor abundant in the individual foraging bees or in honey (Fig 4b). Using presence absence data of of plant and bacterial genera found in different sample types additional plant genera (e.g., *Arabidopsis* and *Prunus*) were found to be present in most honey samples but only rarely in the two types of bee samples and these genera were not observed in flowering vegetation counts (S6 Table and S6 Fig). Visual observation of honey bee visits to flowers captured only a fraction of the associations observed with metabarcoding of the different sample types (S5 Table).

**Fig 5:**
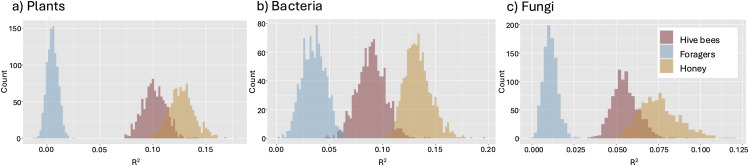
Histograms of R^2^ values of virtual foragers simulated from foragers (blue), virtual foragers simulated from hive bees (red), and virtual foragers simulated from honey (yellow) for a) plants, b) bacteria, and c) fungi.

Our subsampling exercise revealed significant differences in the associations found by each data type. Virtual foragers simulated by subsampling data on hive bees showed significantly different associations with plants, bacteria, and fungi from real foragers (Fig 5 and S7 Table). Similarly, foraging bees did not represent a random subsample of the associations found in honey, with the same pattern repeated across plants, bacteria, and fungi (Fig 5 and S7 Table). Finally, hive bees did not represent a random subsample of the associations found in honey (S7 Fig and S7 Table).

## Discussion

In this study, we investigate whether different sample types yield similar information about honey bees’ associations with plants and with microbes. On top of being important and compelling pollinators, honey bees offer an ideal study organism, since their eusocial lifestyle allows us to sample both bee individuals and the compound record of associations accumulating on several members of the colony or in the honey stored in the hive. By studying each of these levels, we uncovered differences between individuals and colonies and thereby assessed the general impact of sampling methodology in reconstructing associations for broader pollinator communities. Since pollen collected from the surface of individual foraging pollinators is commonly used to reconstruct flower use in pollination ecology [28,29], associations revealed by the singly-collected foraging bees will match the data generated in individually-targeted studies. In fact, our sample size of foraging bees (n = 31 individuals) exceeds the number of individuals typically sampled per species in plant–pollinator networks [52–54].

Based on honey bee biology and the bees’ storage of honey, we *a priori* predicted that individual foraging bees would carry a subsample of the pollen and microbes carried by pooled samples of hive bees. We also expected hive bees, in turn, to carry a subsample of the pollen and microbes found in honey. Our findings refute both expectations as samples of individual foraging bees provide a different impression of honey bee associations with plants and microbes than samples of hive bees or honey. Honey or hive bee sampling are less labor-intensive methods and have a wider scope as the hive bee samples are many individuals and honey captures associations over time, but singly-collected foragers are the sampling method that allows for more direct comparison to other pollinators.

### Associations between honey bees and plants

The lowest richness of plants per sample was recovered from samples of individual forager bees. This was expected as a single individual visits only a subset of the plants visited by the whole colony [55] and is thus likely to encounter fewer plants than a group of pooled bees. Both the honey and hive bee samples combine information from many individual bees. Hive bee samples comprised pools of individuals collected from the same hive, while honey is nectar that is collected and processed by many individual bees [35,36,56]. Therefore, it is also unsurprising that the honey samples showed the highest richness of plant taxa (in terms of ZOTUs or genera) per sample. Likely also because of this joint processing, the community composition of plant DNA in honey was found to be less varied than in the bee sample types.

The set of plant associations recovered from single foraging bees is analogous to sampling methods used for wild pollinators. Encouragingly, the community composition of plants found on individually sampled pollinators did not vary more than sets of plant associations detected on pooled samples of hive bees despite the lower number of associations per individually-sampled bee. Honey, in turn, provides a special case, as it stores microbes and pollen which provide a record of honey bee contact with plants and microbes from the last week and a half (as we collected newly covered honey) or can store old associations from years ago [33]. Unfortunately, this communal archive has no equivalent in most other insects. What should be noted is that no sample type (neither individual foragers, pooled hive bees, nor honey) captured all associations between honey bees and plants, as reconstructed across the full set of sample types. This shows that the different sample types complement each other in reconstructing the full set of associations, as has also been shown for other sampling methods and association types [8,57].

Some of the differences in associations detected between samples of honey and foraging bees will be due to plant phenology. For example, fewer samples of individual foraging bees (20%) than pooled hive bees (58%) or honey (100%) included DNA of willows (*Salix*). The willows flower early in the summer and were only available in low quantities at the time of sampling. The frequent presence of willows in newly covered honey demonstrates that honey provides a fingerprint of the plant DNA collected four to ten days past, as it takes some days to turn nectar into honey and the time needed depends on the weather and the nectar traits [35,36]. Similarly, samples of hive bees, which were made up of individuals of various ages, will likely include young bees that have not begun foraging and thus have only secondary contact with pollen through the honey in the hive and their contact with forager bees. These hive bee samples might also contain bees with honey from the hive in their crops. Such honey might be regurgitated into the tube upon sampling [58]. Thus, while currently flowering plants are likely to occur on singly collected foraging bees and, though secondary contact, on hive bees, recently flowered plants are likely to be detected in hive bees and in honey. Plants with long flowering periods may appear in all three sample types. The latter applies to, e.g., *Anthriscus* and *Taraxacum* in the present dataset, as they generally flower later in the season than *Salix* and were more abundant in our flowering vegetation counts [59]. The time scale represented by the samples will thus vary with the sample type chosen, and the choice of an optimal sampling method will depend on the aims of the study.

### Associations between honey bees and bacteria

Matching the patterns observed for plants, the lowest numbers of bacterial and fungal ZOTUs per sample were recovered from foraging bee samples. However, unlike with plants, the hive bees, and not honey, showed the highest richness of bacterial ZOTUs per sample. Similarly, when comparing bacterial genera, honey did not have significantly higher diversity per sample than did hive bees. Interestingly, the community composition of bacteria detected on samples of foraging bees was more variable than the community composition detected on hive bees or in honey. Foraging bees also had more unique bacterial ZOTUs than either hive bees or honey but these unique ZOTUs, as well as the unique ZOTUs in hive bees and honey had relatively low read abundance, suggesting these may be less common taxa.

We noticed several sample-type specific bacterial trends, including that samples of hive bees all contained bacterial genera commonly associated with the honey bee gut microbiome, such as *Lactobacillus* and *Gilliamella* [23,60]. While some samples of foraging bees also included bee gut bacteria, these samples revealed additional bacteria characteristic of floral microbiomes, such as *Pseudomonas* and *Sphingomonas* [61,62]. The honey samples included nearly as many bacterial genera as did samples of hive bees, including *Lactobacillus* and *Pseudomonas.* Samples of honey also more often contained hive-associated bacteria such as *Apilactobacillus* and *Bombella* [63] (Figs 4 and S6).

While all three types of samples included some representatives of the bee gut microbiome, samples of hive bees contained little else. This suggests that the bees regurgitated or defecated in the tube after sampling. As all the pollen and microbes from the surface of bees were centrifuged and collected for DNA extraction, these excreted gut microbes were then included [64]. As a result, these abundant gut bacteria would then have dominated the amplification process, possibly masking the less abundant external bacteria. The surface bacteria of the bees might then have been under-sampled, and the pool of bacteria detected thus incommensurate to the bacteria detected from the surfaces of the singly collected foraging bees. While we did store the samples immediately on dry ice, the bees sampled in groups may not die immediately as expected because bees can vibrate their wing muscles to generate heat, and when sampled as a group, it is possible the bees could have generated heat to slightly slow the cooling, allowing them to have time to regurgitate or to defecate. To avoid similar biases, we suggest that any samples of hive bees should be killed immediately and singly. Another important distinction between the individual foraging bees and the hive bee sample types that could contribute to the observed differences in bacterial contact is age. Honey bees exhibit age polyethism wherein they perform different tasks as they age, with foraging being their ultimate task [65,66]. Therefore, it is likely that the individual forager samples were older than the bees in the pooled hive bee samples. Given our limited knowledge of the colonization of pollinator surfaces by microbes there is the possibility that age impacts the microbes a honey bee carries. This is an important consideration as the gut microbiome of honey bees, which is more well documented than the surface microbiome, shows age-related changes [67]. Initial gut microbial assembly occurs early in a honey bees’ life within the hive, primarily with socially transmitted taxa and then, as the bee ages, they acquire environmental taxa through foraging [68,69]. Therefore, the increased variability in community composition of bacteria found on foraging bee samples compared to hive bee samples in this study, as well as the increased environmental associated taxa in foraging bee samples, could be age related.

### Associations between honey bees and fungi

Associations with fungi showed a pattern similar to that detected for bacteria, with forager bees carrying fewer fungal ZOTUs than either hive bees or honey. Again, the community composition of fungi detected in samples of foraging bees was more variable than the community composition detected for hive bees and honey. This mirrors what we see in honey bee gut microbiomes where, similar to bacteria, older bees typically have more varied gut fungal communities [70]. Nonetheless, samples of pooled hive bees revealed a higher number of unique fungal ZOTUs than did samples of honey or individual foraging bees. This pattern differed from that detected for plants and bacteria, where hive bees have fewer unique ZOTUs than the foraging bees. This indicates the possibility of differing patterns of acquisition and colonization between bacteria and fungi on pollinator surfaces.

Overall, we were surprised by the finding that samples of individual foraging bees revealed so many taxa not found in samples of pooled hive bees or honey, as we would have expected single bee individuals to carry a smaller diversity than what could be found in the pooled samples. Potentially, on top of age-related differences, the loss of such taxa from the hive interior could be attributed to social immune responses. Honey bee colonies utilize antimicrobial defenses such as propolis envelopes (i.e., plant resins used to coat the entrance and inside of honey bee colonies), which can impact the microbes entering the hive [71,72]. As honey bees also perform extensive allogrooming (i.e., cleaning of each other), some microbes and pollen may be actively removed by hive mates when the foragers enter the hive [73,74].

### Conclusions

Overall, by sampling individual foraging honey bees, pooled honey bees from the hive, and honey, we found that each sampling method captured different associations that honey bees have with plants and microbes. This complementarity illustrates the importance of evaluating the sampling method of pollinator associations, and indicates seasonal, social, and age-related intricacies of honey bee-plant-microbe associations. We do find that sampling individual foraging bees appears to be a good way to sample a large breadth of associations with plants, bacteria, and fungi – with the important caveat that foraging bees did not record associations over as long of a time as the honey stored in the hive. The high number of associations recorded from individual bees seems encouraging in the broader context of pollination networks. Since many wild pollinators are solitary and do not store resources such as honey [75], the large set of associations directly recovered from foraging bees confirms that such point samples offer a comprehensive image of the Eltonian niche of the population. But the sample size of individuals needs to be large, as each individual carries less pollen and fewer microbes than the species is associated with, as shown here by the comparison to honey and hive bee samples. With sufficient numbers, sampling individual pollinators will provide a representative snapshot of the contemporary set of microbes being transported by the bees and the plants being pollinated. Nonetheless, the variability of DNA carried by honey bees as they age and the absence of DNA from plants visited earlier in the season reveals changes in this niche over time. Taken together, our findings thus underscore the need for substantial sample size per species, and repeated sampling over time.

## Supporting information

S1 Fig Comparison of sample read depth and number of ZOTUs of a) plants, b) bacteria, and c) fungi. Each plot has a polynomial regression fit to the data. For plants read depth and ZOTU count do not have a strong relationship, but bacteria and fungi have a read depth under which not all ZOTUs are captured.
(TIF)

S1 Table Resulting p-values of negative binomial general mixed models of the effects of samples reads on ZOTU (plants, bacteria and fungi) and genera (plants and bacteria) count. (DOCX)

S2 Table Mean and standard deviation of number of genera found from different sampling methods across plants, bacteria, and fungi. The log-transformed average number of genera per sampling type are compared with ANOVA and Tukey Post hoc tests, the results of which are presented here.
(DOCX)

S2 Fig Number of hive bees in pooled hive bee samples and the number of ZOTUs that sample yielded of a) plants, b) bacteria, and c) fungi. When a linear regression was added there was no relationship between hive bee number and ZOTU count for plants or microbes (plant adjusted R^2^ = 0.0661, bacteria adjusted R^2^ = −0.0399, fungi adjusted R^2^ = 0.0824).
(TIF)

S3 Fig PCA dispersion diagram of a) plant, b) bacterial, and c) fungal ZOTUs across sample types based on relative read abundances. Foraging bees shown in blue, hive bees shown in red, and honey shown in yellow.
(TIF)

S3 Table Average distance to centroid of PCAs of plants and bacterial genera and statistical comparison of the dispersion from the centroid of the different sample types. Significant *p*-values are shown in bold.
(DOCX)

S4 Fig PCA dispersion diagram of a) plant and b) bacterial genera across sample types. For plants there are no significant differences in dispersion between sample types. For bacterial all sample types have significantly different dispersions from each other. Foraging bees shown in blue, hive bees shown in red, and honey shown in yellow.
(TIF)

S4 Table Average distance to centroid of PCAs of plant, bacterial, and fungal ZOTUs using relative read abundance and statistical comparison of the dispersion from the centroid of the different sample types. Significant *p*-values are shown in bold.
(DOCX)

S5 Fig Euler diagram of a) plant and b) bacterial genera that are found in different sample types. There are similar numbers of overlapping genera across foraging bees, shown in blue, hive bees, shown in red, and honey, shown in yellow both for plants and bacteria.
(TIF)

S6 Fig Plant (a) and bacterial (b) genera found per sample across the different sample types. Among plants, *Anthriscus* (muted green) and *Taraxacum* (pink) were common across all three sample types while *Prunus* (pale blue) and *Arabidopsis* (lime green) were found mainly in honey samples. Among bacteria, hive bee samples were dominated by gut bacteria such as *Lactobacillus* (red), *Gillamella* (purple), and *Snodgrassella* (light brown), while foraging bee and honey samples included a wider variety of bacteria.
(TIF)

S5 Table Plant genera observed using visual and DNA methods and the relative proportion of observations they make up. (DOCX)

S6 Table Flowering vegetation data from the pollinator sampling sites. Family, genus, and species, when known, of observed flowering plants are included, as well as the habitat they were observed in and the counts of the flowers observed.
(DOCX)

S7 Fig Histograms of R^2^ values of mock hive bees simulated from hive bees (red), and mock foragers simulated from honey (gold) for a) plants, b) bacteria, and c) fungi. (TIF)

S7 Table Comparison between simulated and real communities of plant, bacteria, and fungi. Simulated communities used ZOTUs in forager bees, hive bees, and then honey to construct mock foraging bees or hive bees. These were used to compare real forager communities with simulated communities to determine whether associations recovered from foraging bees represent a random subsample of associations recovered from hive bees, and whether associations recovered from hive bees represent a random subsample of associations recovered from honey. Mean R^2^ is the average of 1000 R^2^ values from PERMANOVA comparisons between real and simulated communities. Two sample t-tests were used to compare the simulations and significant *p*-values are shown in bold (Figs 5 and S7 for visualizations).
(DOCX)

S8 Table Sample names for the samples used in this study accessible in the Sequence Read Archive repository, in the BioProject PRJNA1308045 (https://www.ncbi.nlm.nih.gov/sra/PRJNA1308045). (DOCX)
