# Supplementary figures and images for: Inferring plant-bee-microbe associations: Foragers, hive workers, and honey tell complementary stories
Source: PLoS One. 2026 Jul 8;21(7):e0351230. doi: 10.1371/journal.pone.0351230 (PMC13345247; doi:10.1371/journal.pone.0351230)

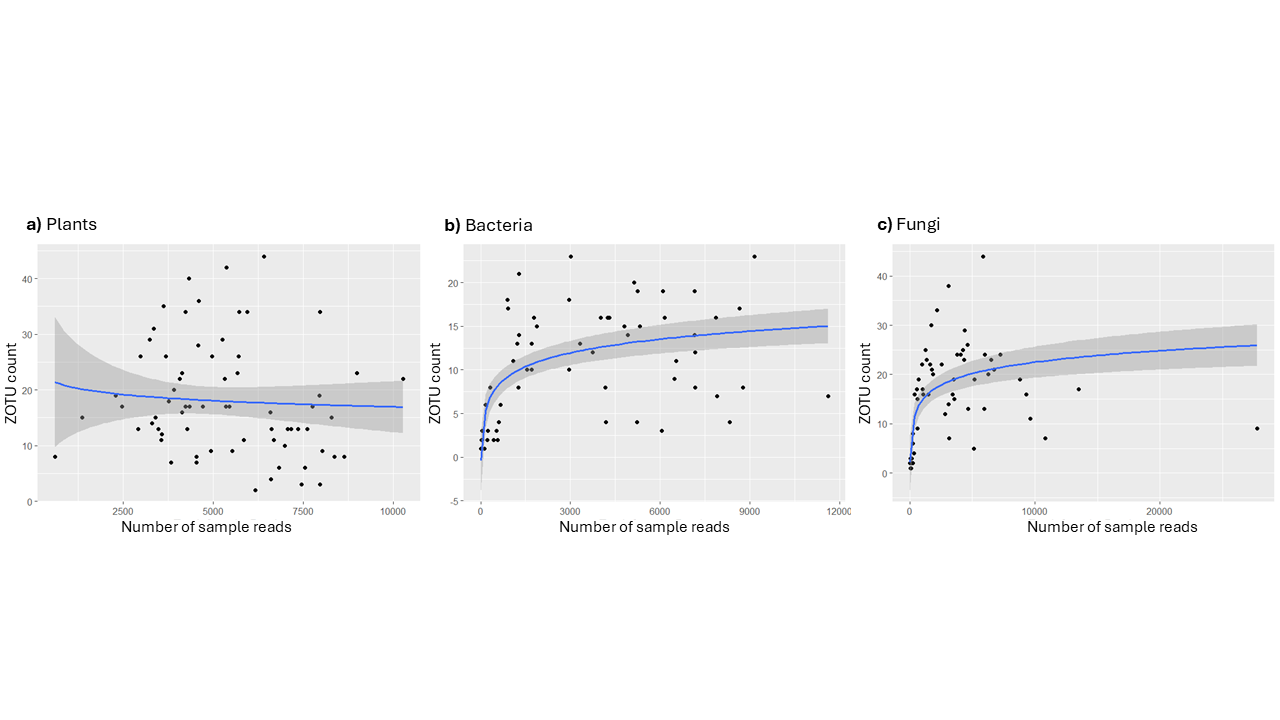

Supplement: S1 Fig — Each plot has a polynomial regression fit to the data. For plants read depth and ZOTU count do not have a strong relationship, but bacteria and fungi have a read depth under which not all ZOTUs are captured. (TIF) [file pone.0351230.s001.tif]

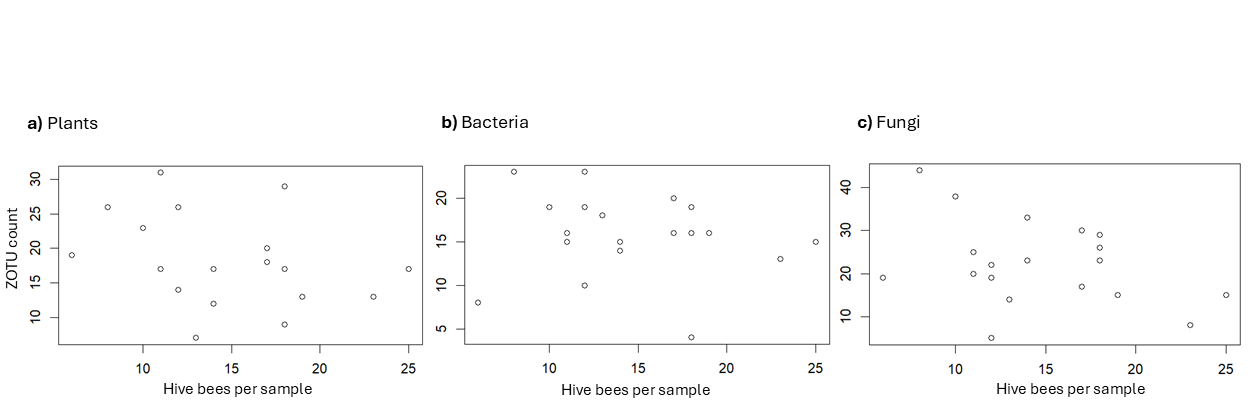

Supplement: S2 Fig — When a linear regression was added there was no relationship between hive bee number and ZOTU count for plants or microbes (plant adjusted R2 = 0.0661, bacteria adjusted R2 = −0.0399, fungi adjusted R2 = 0.0824). (TIF) [file pone.0351230.s004.tif]

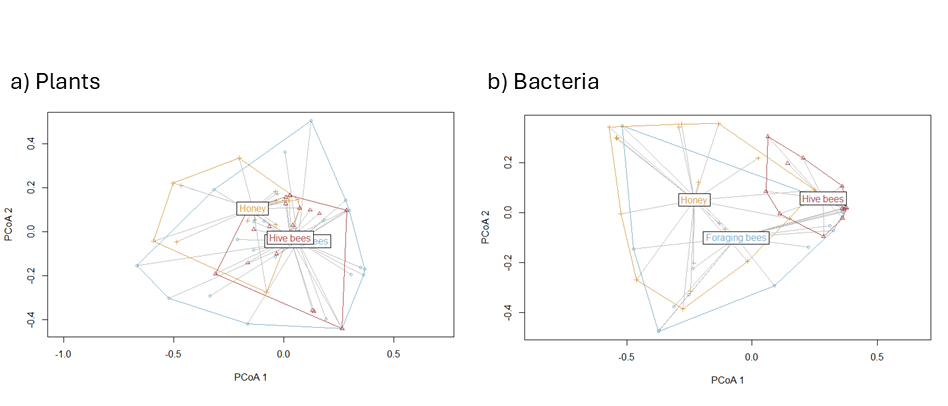

Supplement: S3 Fig — Foraging bees shown in blue, hive bees shown in red, and honey shown in yellow. (TIF) [file pone.0351230.s005.tif]

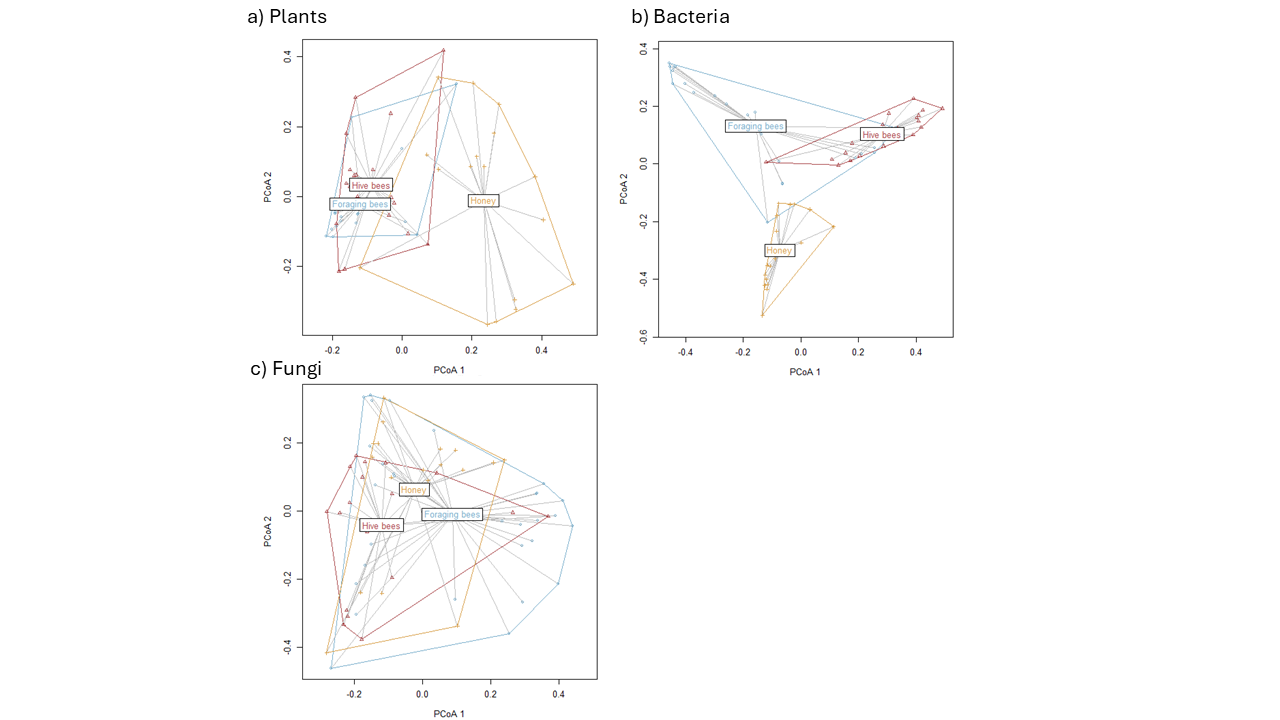

Supplement: S4 Fig — For plants there are no significant differences in dispersion between sample types. For bacterial all sample types have significantly different dispersions from each other. Foraging bees shown in blue, hive bees shown in red, and honey shown in yellow. (TIF) [file pone.0351230.s007.tif]

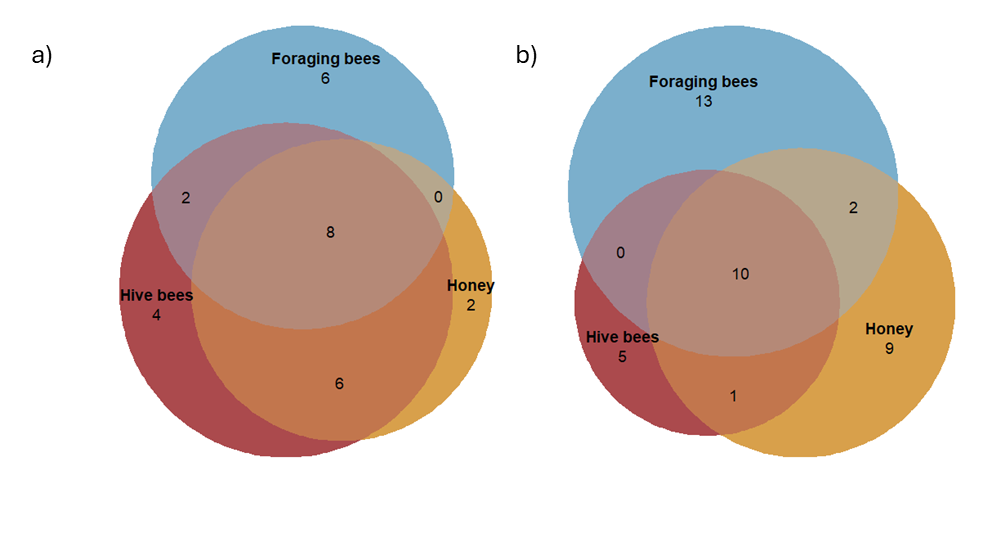

Supplement: S5 Fig — There are similar numbers of overlapping genera across foraging bees, shown in blue, hive bees, shown in red, and honey, shown in yellow both for plants and bacteria. (TIF) [file pone.0351230.s009.tif]

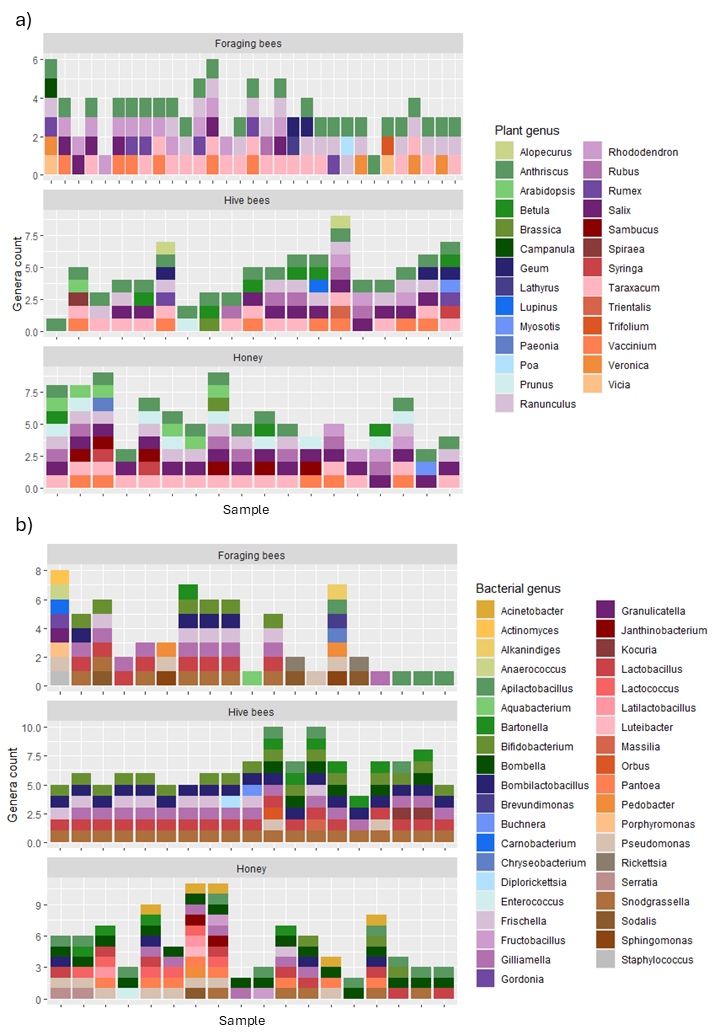

Supplement: S6 Fig — Among plants, Anthriscus (muted green) and Taraxacum (pink) were common across all three sample types while Prunus (pale blue) and Arabidopsis (lime green) were found mainly in honey samples. Among bacteria, hive bee samples were dominated by gut bacteria such as Lactobacillus (red), Gillamella (purple), and Snodgrassella (light brown), while foraging bee and honey samples included a wider variety of bacteria. (TIF) [file pone.0351230.s010.tif]

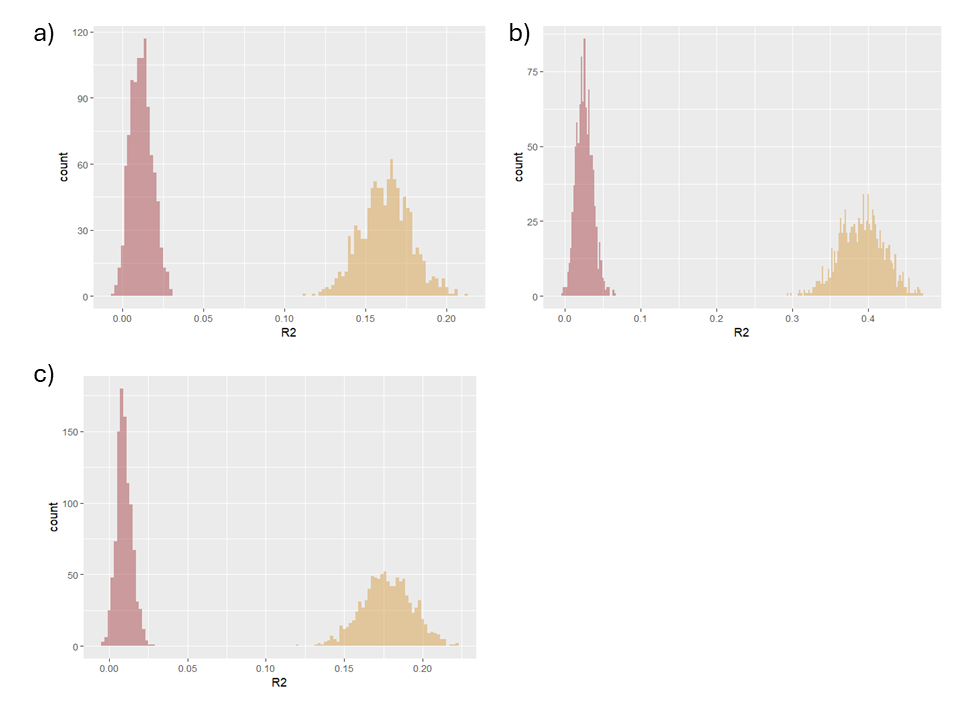

Supplement: S7 Fig — (TIF) [file pone.0351230.s013.tif]
