# Supplementary material for: Inferring plant-bee-microbe associations: Foragers, hive workers, and honey tell complementary stories
Source: PLoS One. 2026 Jul 8;21(7):e0351230. doi: 10.1371/journal.pone.0351230 (PMC13345247; doi:10.1371/journal.pone.0351230)
Supplement: S1 Table — (DOCX) [file pone.0351230.s002.docx]

|  | Plants | | Bacteria | | Fungi |
| --- | --- | --- | --- | --- | --- |
|  | ZOTU | Genera | ZOTU | Genera | ZOTU |
| Sample reads | 0.458 | 0.153 | **<0.001** | **<0.001** | **<0.001** |
