# Supplementary material for: Inferring plant-bee-microbe associations: Foragers, hive workers, and honey tell complementary stories
Source: PLoS One. 2026 Jul 8;21(7):e0351230. doi: 10.1371/journal.pone.0351230 (PMC13345247; doi:10.1371/journal.pone.0351230)
Supplement: S2 Table — The log-transformed average number of genera per sampling type are compared with ANOVA and Tukey Post hoc tests, the results of which are presented here. (DOCX) [file pone.0351230.s003.docx]

|  | Sample type | Mean±SD | ANOVA | Comparison | Tukey HSD  *p*-value |
| --- | --- | --- | --- | --- | --- |
| Plants | Foraging bees | 3.48±1.18 | f-value: 7.94  *p*-value: **<0.001** | Foraging vs. Hive bees | 0.092 |
|  | Hive bees | 4.68±1.92 |  | Foraging bees vs. Honey | **<0.001** |
|  | Honey | 5.67±1.97 |  | Hive bees vs. Honey | 0.235 |
| Bacteria | Foraging bees | 3.58±2.50 | f-value: 10.49  *p*-value: **<0.001** | Foraging vs. Hive bees | **<0.001** |
|  | Hive bees | 6.42±1.61 |  | Foraging bees vs. Honey | **0.009** |
|  | Honey | 5.56±2.85 |  | Hive bees vs. Honey | 0.400 |
