# Supplementary material for: Inferring plant-bee-microbe associations: Foragers, hive workers, and honey tell complementary stories
Source: PLoS One. 2026 Jul 8;21(7):e0351230. doi: 10.1371/journal.pone.0351230 (PMC13345247; doi:10.1371/journal.pone.0351230)
Supplement: S3 Table — Significant p-values are shown in bold. (DOCX) [file pone.0351230.s006.docx]

|  | Sample Type | Average distance to centroid | Comparison | Dispersion *p*-value |
| --- | --- | --- | --- | --- |
| Plants | Foraging bees | 0.369 | Foraging vs. hive bees | 0.734 |
|  | Hive bees | 0.352 | Foraging bees vs. honey | 0.366 |
|  | Honey | 0.306 | Hive bees vs. honey | 0.366 |
| Bacteria | Foraging bees | 0.603 | Foraging vs. hive bees | **0.001** |
|  | Hive bees | 0.175 | Foraging bees vs. honey | **0.001** |
|  | Honey | 0.314 | Hive bees vs. honey | **0.001** |
