# Supplementary material for: Inferring plant-bee-microbe associations: Foragers, hive workers, and honey tell complementary stories
Source: PLoS One. 2026 Jul 8;21(7):e0351230. doi: 10.1371/journal.pone.0351230 (PMC13345247; doi:10.1371/journal.pone.0351230)
Supplement: S4 Table — Significant p-values are shown in bold. (DOCX) [file pone.0351230.s008.docx]

|  | Comparison | Dispersion *p*-value | Dispersion F-stat | PERMANOVA *p*-value |
| --- | --- | --- | --- | --- |
| Plants | Foraging vs. hive bees | 0.462 | F-stat 4.09 | *p*-value **0.001**  F-stat 3.67 |
|  | Foraging bees vs. honey | **0.027** |  |  |
|  | Hive bees vs. honey | 0.075 |  |  |
| Bacteria | Foraging vs. hive bees | 0.233 | F-stat 7.98 | *p*-value **0.001**  F-stat 6.99 |
|  | Foraging bees vs. honey | **0.014** |  |  |
|  | Hive bees vs. honey | **0.002** |  |  |
| Fungi | Foraging vs. hive bees | **0.003** | F-stat 20.88 | *p*-value **0.001**  F-stat 2.903 |
|  | Foraging bees vs. honey | **0.002** |  |  |
|  | Hive bees vs. honey | **0.002** |  |  |
