# Supplementary material for: Inferring plant-bee-microbe associations: Foragers, hive workers, and honey tell complementary stories
Source: PLoS One. 2026 Jul 8;21(7):e0351230. doi: 10.1371/journal.pone.0351230 (PMC13345247; doi:10.1371/journal.pone.0351230)
Supplement: S5 Table — (DOCX) [file pone.0351230.s011.docx]

| **Genus** | **Visual observation** | **Foraging bees** | **Hive bees** | **Honey** | **Combined** |
| --- | --- | --- | --- | --- | --- |
| *Alopecurus* | 0 | 0 | 0.002 | 0 | 0.0005 |
| *Anthriscus* | 0.069 | 0.147 | 0.349 | 0.092 | 0.176 |
| *Arabidopsis* | 0 | 0 | 0.0005 | 0.027 | 0.006 |
| *Betula* | 0 | 0 | 0.110 | 0.006 | 0.023 |
| *Brassica* | 0 | 0 | 0.003 | 0.002 | 0.001 |
| *Campanula* | 0 | 0.00006 | 0 | 0 | 0.00003 |
| *Geum* | 0 | 0.0005 | 0.011 | 0 | 0.002 |
| *Lathyrus* | 0 | 0.0002 | 0 | 0 | 0.0004 |
| *Lupinus* | 0 | 0 | 0.0008 | 0 | 0.0002 |
| *Myosotis* | 0 | 0 | 0.0002 | 0.085 | 0.019 |
| *Paeonia* | 0 | 0 | 0 | 0.0003 | 0.00005 |
| *Poa* | 0 | 0.0001 | 0 | 0 | 0.00008 |
| *Prunus* | 0 | 0 | 0.002 | 0.022 | 0.005 |
| *Ranunculus* | 0.035 | 0.091 | 0.164 | 0.023 | 0.091 |
| *Rhododendron* | 0.241 | 0.221 | 0.016 | 0.049 | 0.142 |
| *Rubus* | 0 | 0.00005 | 0.145 | 0.009 | 0.031 |
| *Rumex* | 0 | 0.035 | 0.004 | 0 | 0.021 |
| *Salix* | 0 | 0.008 | 0.054 | 0.573 | 0.142 |
| *Sambucus* | 0 | 0 | 0 | 0.006 | 0.001 |
| *Spiraea* | 0 | 0 | 0.001 | 0 | 0.0003 |
| *Syringa* | 0 | 0 | 0.0002 | 0.0003 | 0.0001 |
| *Taraxacum* | 0.621 | 0.457 | 0.118 | 0.102 | 0.310 |
| *Trientalis* | 0 | 0 | 0.0004 | 0 | 0.00008 |
| *Trifolium* | 0 | 0.0009 | 0 | 0 | 0.0005 |
| *Vaccinium* | 0.035 | 0.013 | 0.018 | 0.003 | 0.012 |
| *Veronica* | 0 | 0.019 | 0 | 0 | 0.011 |
| *Vicia* | 0 | 0.006 | 0 | 0 | 0.004 |
