# Supplementary material for: Inferring plant-bee-microbe associations: Foragers, hive workers, and honey tell complementary stories
Source: PLoS One. 2026 Jul 8;21(7):e0351230. doi: 10.1371/journal.pone.0351230 (PMC13345247; doi:10.1371/journal.pone.0351230)
Supplement: S6 Table — Family, genus, and species, when known, of observed flowering plants are included, as well as the habitat they were observed in and the counts of the flowers observed. (DOCX) [file pone.0351230.s012.docx]

| **Family** | **Genus** | **Species** | **Habitat** | **Count** |
| --- | --- | --- | --- | --- |
| Apiaceae | *Anthriscus* | *sylvestris* | Field | 12735 |
| Asteraceae | *Taraxacum* | *sp* | Field | 571 |
|  | *Achillea* | *millefolium* | Field | 10 |
|  | *Hierarchium* | *sp* | Field | 233 |
| Brassicaceae | *Barbarea* | *vulgaris* | Field | 7 |
| Campanulaceae | *Campanula* | *rotundifolia* | Field | 411 |
| Caryophyllaceae | *Stellaria* | *graminea* | Field | 781 |
|  | *Cerastium* | *fontanum* | Field | 3 |
| Ericaceae | *Andromena* | *polifolia* | Forest | 1 |
|  | *Rhododendron* | *tomentosum* | Forest | 2600 |
|  | *Vaccinium* | *myrtillus* | Forest | 520 |
|  |  | *uliginosum* | Forest | 1295 |
|  |  | *vitis-idaea* | Forest | 522 |
| Fabaceae | *Lathyrus* | *pratensis* | Field | 23 |
|  | *Vicia* | *cracca* | Field | 25 |
| Plantaginaceae | *Veronica* | *chamaedrys* | Field | 860 |
| Primulaceae | *Lysimachia* | *europaea* | Forest | 27 |
| Ranunculacea | *Ranunculus* | *sp* | Field | 11203 |
| Rosaceae | *Alchemilla* | *sp* | Field | 130 |
|  | *Fragaria* | *vesca* | Field | 90 |
|  | *Geum* | *rivale* | Field | 76 |
|  | *Rubus* | *arcticus* | Field | 21 |
|  | *Sorbus* | *aucuparia* | Field | 40 |
| Rubiaceae | *Galium* | *uliginosum* | Field | 1 |
| Salicaceae | *Salix* | *sp* | Forest | 52 |
| Violaceae | *Viola* | *canina* | Field | 10 |
