# Supplementary material for: Inferring plant-bee-microbe associations: Foragers, hive workers, and honey tell complementary stories
Source: PLoS One. 2026 Jul 8;21(7):e0351230. doi: 10.1371/journal.pone.0351230 (PMC13345247; doi:10.1371/journal.pone.0351230)
Supplement: S7 Table — Simulated communities used ZOTUs in forager bees, hive bees, and then honey to construct mock foraging bees or hive bees. These were used to compare real forager communities with simulated communities to determine whether associations recovered from foraging bees represent a random subsample of associations recovered from hive bees, and whether associations recovered from hive bees represent a random subsample of associations recovered from honey. Mean R2 is the average of 1000 R2 values from PERMANOVA comparisons between real and simulated communities. Two sample t-tests were used to compare the simulations and significant p-values are shown in bold (Figs 5 and S7 for visualizations). (DOCX) [file pone.0351230.s014.docx]

|  |  | Mean R2 | *p*-value when compared to foragers simulated from foragers |
| --- | --- | --- | --- |
| Foraging bees simulated from foraging bees vs. real foragers | Plants | 0.0054 | NA |
|  | Bacteria | 0.0352 | NA |
|  | Fungi | 0.0110 | NA |
| Foraging bees simulated from hive bees vs. real foragers | Plants | 0.1006 | **<0.001** |
|  | Bacteria | 0.0889 | **<0.001** |
|  | Fungi | 0.0559 | **<0.001** |
| Foraging bees simulated from honey vs. real foragers | Plants | 0.1269 | **<0.001** |
|  | Bacteria | 0.1334 | **<0.001** |
|  | Fungi | 0.0754 | **<0.001** |
| Hive bees simulated from hive bees vs. real hive bees | Plants | 0.0116 | NA |
|  | Bacteria | 0.0260 | NA |
|  | Fungi | 0.0096 | NA |
| Hive bees simulated from honey vs. real hive bees | Plants | 0.1626 | **<0.001** |
|  | Bacteria | 0.3914 | **<0.001** |
|  | Fungi | 0.1773 | **<0.001** |
