# Supplementary material for: Inferring plant-bee-microbe associations: Foragers, hive workers, and honey tell complementary stories
Source: PLoS One. 2026 Jul 8;21(7):e0351230. doi: 10.1371/journal.pone.0351230 (PMC13345247; doi:10.1371/journal.pone.0351230)
Supplement: S8 Table — (DOCX) [file pone.0351230.s015.docx]

| Plant data | Bacterial data | Fungal data |
| --- | --- | --- |
| ITpBEE0070 | 16sBEE0070 | ITfBEE0070 |
| ITpBEE0116 | 16sBEE0116 | ITfBEE0116 |
| ITpBEE0253 | 16sBEE0253 | ITfBEE0253 |
| ITpBEE0384 | 16sBEE0384 | ITfBEE0384 |
| ITpBEE0430 | 16sBEE0430 | ITfBEE0430 |
| ITpBEE0495 | 16sBEE0495 | ITfBEE0495 |
| ITpBEE0496 | 16sBEE0496 | ITfBEE0496 |
| ITpBEE0497 | 16sBEE0497 | ITfBEE0497 |
| ITpBEE0498 | 16sBEE0498 | ITfBEE0498 |
| ITpBEE0499 | 16sBEE0499 | ITfBEE0499 |
| ITpBEE0500 | 16sBEE0500 | ITfBEE0500 |
| ITpBEE0501 | 16sBEE0501 | ITfBEE0501 |
| ITpBEE0502 | 16sBEE0502 | ITfBEE0502 |
| ITpBEE0503 | 16sBEE0503 | ITfBEE0503 |
| ITpBEE0504 | 16sBEE0504 | ITfBEE0504 |
| ITpBEE0505 | 16sBEE0505 | ITfBEE0505 |
| ITpBEE0506 | 16sBEE0506 | ITfBEE0506 |
| ITpBEE0507 | 16sBEE0507 | ITfBEE0507 |
| ITpBEE0508 | 16sBEE0508 | ITfBEE0508 |
| ITpBEE0509 | 16sBEE0509 | ITfBEE0509 |
| ITpBEE0510 | 16sBEE0510 | ITfBEE0510 |
| ITpBEE0511 | 16sBEE0511 | ITfBEE0511 |
| ITpBEE0512 | 16sBEE0512 | ITfBEE0512 |
| ITpBEE0513 | 16sBEE0513 | ITfBEE0513 |
| ITpBEE0477 | 16sBEE0477 | ITfBEE0477 |
| ITpBEE0478 | 16sBEE0478 | ITfBEE0478 |
| ITpBEE0479 | 16sBEE0479 | ITfBEE0479 |
| ITpBEE0480 | 16sBEE0480 | ITfBEE0480 |
| ITpBEE0481 | 16sBEE0481 | ITfBEE0481 |
| ITpBEE0482 | 16sBEE0482 | ITfBEE0482 |
| ITpBEE0483 | 16sBEE0483 | ITfBEE0483 |
| ITpBEE0484 | 16sBEE0484 | ITfBEE0484 |
| ITpBEE0485 | 16sBEE0485 | ITfBEE0485 |
| ITpBEE0486 | 16sBEE0486 | ITfBEE0486 |
| ITpBEE0487 | 16sBEE0487 | ITfBEE0487 |
| ITpBEE0488 | 16sBEE0488 | ITfBEE0488 |
| ITpBEE0489 | 16sBEE0489 | ITfBEE0489 |
| ITpBEE0490 | 16sBEE0490 | ITfBEE0490 |
| ITpBEE0491 | 16sBEE0491 | ITfBEE0491 |
| ITpBEE0492 | 16sBEE0492 | ITfBEE0492 |
| ITpBEE0493 | 16sBEE0493 | ITfBEE0493 |
| ITpBEE0494 | 16sBEE0494 | ITfBEE0494 |
| ITpBEE0328 | 16sBEE0328 | ITfBEE0328 |
| ITpBEE0013C | 16sBEE0013C | ITfBEE0013C |
| ITpBEE0031 | 16sBEE0031 | ITfBEE0031 |
| ITpBEE0076 | 16sBEE0076 | ITfBEE0076 |
| ITpBEE0080 | 16sBEE0080 | ITfBEE0080 |
| ITpBEE0085 | 16sBEE0085 | ITfBEE0085 |
| ITpBEE0101 | 16sBEE0101 | ITfBEE0101 |
| ITpBEE0106 | 16sBEE0106 | ITfBEE0106 |
| ITpBEE0112 | 16sBEE0112 | ITfBEE0112 |
| ITpBEE0119 | 16sBEE0119 | ITfBEE0119 |
| ITpBEE0120 | 16sBEE0120 | ITfBEE0120 |
| ITpBEE0121 | 16sBEE0121 | ITfBEE0121 |
| ITpBEE0125 | 16sBEE0125 | ITfBEE0125 |
| ITpBEE0126 | 16sBEE0126 | ITfBEE0126 |
| ITpBEE0128 | 16sBEE0128 | ITfBEE0128 |
| ITpBEE0129 | 16sBEE0129 | ITfBEE0129 |
| ITpBEE0173 | 16sBEE0173 | ITfBEE0173 |
| ITpBEE0209 | 16sBEE0209 | ITfBEE0209 |
| ITpBEE0223 | 16sBEE0223 | ITfBEE0223 |
| ITpBEE0262 | 16sBEE0262 | ITfBEE0262 |
| ITpBEE0350 | 16sBEE0350 | ITfBEE0350 |
| ITpBEE0375 | 16sBEE0375 | ITfBEE0375 |
| ITpBEE0433 | 16sBEE0433 | ITfBEE0433 |
| ITpBEE0434 | 16sBEE0434 | ITfBEE0434 |
| ITpBEE0440 | 16sBEE0440 | ITfBEE0440 |
| ITpBEE0448 | 16sBEE0448 | ITfBEE0448 |
